# Imetelstat as a novel therapeutic approach for myelodysplastic syndrome

**DOI:** 10.1016/j.htct.2024.09.2484

**Published:** 2025-02-11

**Authors:** Ammara Waheed Arain, Anushka Andani, Aayat Kashif, Radeyah Waseem

**Affiliations:** Liaquat National Hospital, Karachi, Pakistan

Dear Editor,

Myelodysplastic syndrome (MDS) is a conglomerate of hematologic diseases characterized by dysplasia of hematopoietic stem cells and ineffective hematopoiesis in the bone marrow, causing variable degrees of peripheral cytopenia. MDS is associated with an increased probability of developing acute myeloid leukemia (AML).^1^ Prognostic scoring systems divide MDS into low, intermediate, and high-risk categories, where the likelihood of encountering AML is greatest in the high-risk category. The following factors contribute to high-risk classifications: percentage of bone marrow blasts >10 %, severe cytopenia, and Revised International Prognostic Scoring System (IPSS-R) score >4.5. Intermediate and low-risk MDS have moderate to low risk of developing AML and a better overall prognosis. Factors include intermediate (5–9 %) to low percentage of bone marrow blasts (<5 %), mild to moderate cytopenia, and IPSS-R scores (3–4.5) and <3, respectively.^1^

The treatment goal is to decrease the risk of leukemic progression achieved by stem cell transplantation, which is only recommended if patients are suitable for intervention. Patients not suitable for transplant are treated with hypomethylating agents such as azacitidine or decitabine (Dacogen). In low-risk MDS, the treatment goal is to manage anemia and other cytopenia and their associated manifestations. Treatment modalities in such candidates include hematopoietic growth factors, luspatercept in candidates with ring sideroblasts, epigenetic modifiers, erythrocyte transfusions, and immunosuppressive drugs.^1^

Imetelstat was approved for the treatment of MDS with erythrocyte transfusion anemia by the Food and Drugs Administration of the USA (FDA) on 6th June 2024.^2^

Imetelstat is a 13-mer oligonucleotide that binds to the RNA template of telomerase to inhibit it.^3^ This causes malignant hematopoietic progenitor cells that exhibit high telomerase tendency to undergo apoptosis while sparing normal cells. This process aids in the restoration of erythropoiesis and bone marrow function.^3^ In MDS, the bone marrow is dominated by malignant clones that are defective and have shorter telomere length and enhanced telomerase activity resulting in inefficient erythropoiesis. Therefore, telomerase inhibition presents a viable treatment approach for MDS.^3^

In the Phase-II study, imetelstat therapy resulted in long-term, continuous transfusion independence in intensively transfused patients with low-risk MDS.^4^ The IMerge Phase III study demonstrated enhanced transfusion independence, with 39.8 % attaining independence at eight weeks versus 15 % on placebo; at 24 wk the rates were 28 % versus 3.3 % with manageable safety profiles, pointing to imetelstat's promise in low-risk MDS. ^3^ In contrast to JAK inhibitors, imetelstat is a first-in-class telomerase inhibitor, that has demonstrated long-lasting responses in myeloproliferative neoplasms (MPNs), specifically those with essential thrombocythemia.^3^ It has improved overall survival in refractory/relapsed myelofibrosis patients, indicating its potential as a feasible treatment option with manageable side effects.^3^

Imetelstat has demonstrated blood-related and non-blood-related side effects. Blood-related side effects include thrombocytopenia and neutropenia, while non-blood-related side effects include weakness, tiredness, COVID-19, leg swelling, headache, diarrhea, fatigue, arthralgia, myalgia, hypertension, dyspnea, nausea, asthenia, dizziness, sepsis, fracture, heart failure, hemorrhage, and other fatal adverse reactions.^5^ Certain limitations of imetelstat therapy for lower-risk MDS are off-site effects other than telomerase inhibition: shortage of known biomarkers like the initial telomere length was unknown, effects on normal hematopoietic stem cells like decreased normal CD34^+^ cells observed in mouse models,^6^ restricted data on long term effects and restricted comparative data. There are numerous future implications of imetelstat therapy besides benefits in treating patients with lower-risk MDS and other hemolytic malignancies. Currently, Geron is conducting a Phase III clinical trial of imetelstat in JAK-inhibitor relapsed/refractory myelofibrosis (R/R MF). This medication is also being considered as a potential treatment for other hemolytic malignancies. While imetelstat is being used as a treatment for patients with myelofibrosis after its efficacy was proved by clinical trials like the IMbark Trial, and IMpactMF Trial.^7^

## Conflicts of interest

None.
